# Effect of ten-valent pneumococcal conjugate vaccine on invasive pneumococcal disease and nasopharyngeal carriage in Kenya: a longitudinal surveillance study

**DOI:** 10.1016/S0140-6736(18)33005-8

**Published:** 2019-05-25

**Authors:** Laura L Hammitt, Anthony O Etyang, Susan C Morpeth, John Ojal, Alex Mutuku, Neema Mturi, Jennifer C Moisi, Ifedayo M Adetifa, Angela Karani, Donald O Akech, Mark Otiende, Tahreni Bwanaali, Jackline Wafula, Christine Mataza, Edward Mumbo, Collins Tabu, Maria Deloria Knoll, Evasius Bauni, Kevin Marsh, Thomas N Williams, Tatu Kamau, Shahnaaz K Sharif, Orin S Levine, J Anthony G Scott

**Affiliations:** a Epidemiology and Demography Department, KEMRI-Wellcome Trust Research Programme, Centre for Geographic Medicine-Coast, Kilifi, Kenya; b Nuffield Department of Clinical Medicine, University of Oxford, Oxford, UK; c Department of International Health, Johns Hopkins Bloomberg School of Public Health, Baltimore, MD, USA; d Department of Infectious Disease Epidemiology, London School of Hygiene & Tropical Medicine, London, UK; e Pfizer Vaccines, Paris, France; f County Health Department, Kilifi County, Kenya; g National Vaccines and Immunization Programme, Ministry of Health, Kenya; h Imperial College, London, UK; i INDEPTH Network, Accra, Ghana; j Bill & Melinda Gates Foundation, Seattle, WA, USA

## Abstract

**Background:**

Ten-valent pneumococcal conjugate vaccine (PCV10), delivered at 6, 10, and 14 weeks of age was introduced in Kenya in January, 2011, accompanied by a catch-up campaign in Kilifi County for children aged younger than 5 years. Coverage with at least two PCV10 doses in children aged 2–11 months was 80% in 2011 and 84% in 2016; coverage with at least one dose in children aged 12–59 months was 66% in 2011 and 87% in 2016. We aimed to assess PCV10 effect against nasopharyngeal carriage and invasive pneumococcal disease (IPD) in children and adults in Kilifi County.

**Methods:**

This study was done at the KEMRI-Wellcome Trust Research Programme among residents of the Kilifi Health and Demographic Surveillance System, a rural community on the Kenyan coast covering an area of 891 km^2^. We linked clinical and microbiological surveillance for IPD among admissions of all ages at Kilifi County Hospital, Kenya, which serves the community, to the Kilifi Health and Demographic Surveillance System from 1999 to 2016. We calculated the incidence rate ratio (IRR) comparing the prevaccine (Jan 1, 1999–Dec 31, 2010) and postvaccine (Jan 1, 2012–Dec 31, 2016) eras, adjusted for confounding, and reported percentage reduction in IPD as 1 minus IRR. Annual cross-sectional surveys of nasopharyngeal carriage were done from 2009 to 2016.

**Findings:**

Surveillance identified 667 cases of IPD in 3 211 403 person-years of observation. Yearly IPD incidence in children younger than 5 years reduced sharply in 2011 following vaccine introduction and remained low (PCV10-type IPD: 60·8 cases per 100 000 in the prevaccine era *vs* 3·2 per 100 000 in the postvaccine era [adjusted IRR 0·08, 95% CI 0·03–0·22]; IPD caused by any serotype: 81·6 per 100 000 *vs* 15·3 per 100 000 [0·32, 0·17–0·60]). PCV10-type IPD also declined in the post-vaccination era in unvaccinated age groups (<2 months [no cases in the postvaccine era], 5–14 years [adjusted IRR 0·26, 95% CI 0·11–0·59], and ≥15 years [0·19, 0·07–0·51]). Incidence of non-PCV10-type IPD did not differ between eras. In children younger than 5 years, PCV10-type carriage declined between eras (age-standardised adjusted prevalence ratio 0·26, 95% CI 0·19–0·35) and non-PCV10-type carriage increased (1·71, 1·47–1·99).

**Interpretation:**

Introduction of PCV10 in Kenya, accompanied by a catch-up campaign, resulted in a substantial reduction in PCV10-type IPD in children and adults without significant replacement disease. Although the catch-up campaign is likely to have brought forward the benefits by several years, the study suggests that routine infant PCV10 immunisation programmes will provide substantial direct and indirect protection in low-income settings in tropical Africa.

**Funding:**

Gavi, The Vaccine Alliance and The Wellcome Trust of Great Britain.

## Introduction

The number of pneumococcal deaths in children aged 1–59 months was estimated at 317 000 globally in 2015, a decline of more than 50% from 2000.^1^ In middle-income and high-income countries, inclusion of pneumococcal conjugate vaccines (PCVs) in routine infant vaccination programmes has led to a substantial reduction in the incidence of invasive pneumococcal disease (IPD) caused by vaccine serotypes (VTs). In addition, because vaccinated children are less likely to carry and transmit VT pneumococci, programmatic use of vaccination in children has resulted in a decline in IPD in unvaccinated individuals (ie, herd protection).^2^ Evidence of the effect of PCVs in low- income settings is sparse and effect models rely on efficacy estimates from randomised controlled trials, not real-world implementation. Furthermore, there is no evidence of the effect of ten-valent PCV (PCV10) conjugated to non-typeable *Haemophilus influenzae* on invasive pneumococcal disease in Africa, where the greatest burden of deaths from pneumococcal disease occur.

61% (82·4 million) of the world's infants have not received PCV; however, dozens of low-income countries have introduced PCV or will do so in the coming decade.^3^ In 2011, with support from Gavi, the Vaccine Alliance, Kenya became one of the first countries in Africa to introduce PCV and the first country in Africa to use PCV10. PCV10 was introduced in the Kenyan national childhood immunisation schedule as a three-dose series administered at 6, 10, and 14 weeks of age. There is good evidence of the efficacy of PCV9 in African settings, but efficacy of PCV10 (Synflorix; GlaxoSmithKline) has not been shown.^4^, ^5^ Furthermore, because of the potential for substantial herd protection or for serotype replacement disease, the net population benefit of the PCV programme in these settings can only be estimated through longitudinal IPD surveillance. This information is essential to support realistic cost-effectiveness analyses and sustain the commitment of Ministries of Health to the PCV programme as countries transition from Gavi support for vaccines to self-financing.

**Research in context**

**Evidence before this study**

In middle-income and high-income countries, inclusion of pneumococcal conjugate vaccines (PCVs) in routine infant vaccination programmes has led to a substantial reduction in the incidence of invasive pneumococcal disease (IPD) caused by vaccine serotypes. However, pneumococcal disease remains a leading vaccine-preventable cause of childhood mortality and most of these deaths occur in Africa. This study was planned in 2006, after Gavi, the Vaccine Alliance, made the decision to support introduction of PCVs in lower-income countries. It aimed to capture the population effect of PCV introduction in operational use against IPD and nasopharyngeal carriage. Although there are data from The Gambia showing a reduction in IPD in young children 5 years after the introduction of seven-valent pneumococcal conjugate vaccine and 3 years following the introduction of 13-valent pneumococcal conjugate vaccine, there are no effect data from low-income settings that have adopted ten-valent pneumococcal conjugate vaccine (PCV10). In addition, the duration of the study in The Gambia was not sufficient to assess the indirect effects of the PCV programme. Establishing the population effect of PCV in low-resource settings is essential to sustain the commitment of Ministries of Health to PCV programmes.

**Added value of this study**

This study provides the first population-level evidence of the effect of a PCV10 programme in a low-income country. In this 18-year population-based, laboratory-based surveillance study, introduction of PCV10 with a catch-up campaign for children under 5 years and without a booster dose reduced the incidence of PCV10-type IPD in children aged up to 5 years (by 92% [95% CI 78–97]) and in unvaccinated age groups (74% [41–89] in the 5–14-year age group; 81% [49–93] in the 15 years and older age group). There was no significant change in the incidence of non-PCV10-type IPD. The observed decline in PCV10-type IPD is supported by the observed trends in vaccine-type pneumococcal carriage.

**Implications of all the available evidence**

Our study suggests that use of PCV10 in low-income settings will lead to substantial health benefits for the whole population, not just vaccine recipients. These results will underpin policy making in African countries as they confront the challenge of continuing PCV programmes independently of Gavi.

Through a collaboration between the Kenyan Ministry of Health, Gavi, and the KEMRI-Wellcome Trust Research Programme (KWTRP), we used an existing integrated demographic, clinical, and microbiological surveillance system to do a prospectively designed assessment of vaccine effect against nasopharyngeal carriage and IPD in children and adults before and after introduction of PCV10 in the routine infant immunisation programme in Kenya.

## Methods

### Study design and participants

This study was done at KWTRP among residents of the Kilifi Health and Demographic Surveillance System (KHDSS), a rural community on the Kenyan coast covering an area of 891 km^2^. A census of the KHDSS in 2000 defined the resident population, and all subsequent births, deaths, and migration events were monitored by fieldworker visits to every participating household at approximately 4-monthly intervals.^6^ The population (179 568 in 1999; 239 392 in 2007; 284 826 in 2016) is served by a single government hospital, Kilifi County Hospital (KCH). Among women attending antenatal clinic at KCH, the prevalence of HIV infection ranged between 2·1% and 4·6% during 2005–16 (appendix). The prevalence of HIV among children aged up to 5 years in Kenya was estimated in 2012 at 1·6%.^7^ *H influenzae* type b conjugate vaccine was introduced in Kenya in 2001.^8^ In the 12 years before PCV10 introduction, the serotypes contained in PCV10 (1, 4, 5, 6B, 7F, 9V, 14, 18C, 19F, and 23F) comprised 75% of IPD in children aged up to 5 years in Kilifi. The protocol was approved by the Oxford Tropical Ethical Review Committee (No. 30-10) and the Kenya National Ethical Review Committee (SSC1433). Adult participants and parents or guardians of all child participants provided written informed consent.

### Procedures

In January, 2011, the Government of Kenya introduced PCV10 into the national immunisation schedule, administered simultaneously with pentavalent vaccine (diphtheria–whole cell pertussis–tetanus–hepatitis B–*H influenzae* type b vaccine) at 6, 10, and 14 weeks of age. A national catch-up campaign provided three doses of PCV10 to children aged less than 12 months. As part of the study design, the Ministry of Health did a catch-up campaign in Kilifi County providing up to two doses of PCV10 to children aged 12–59 months in two campaigns, beginning on Jan 31, 2011 and March 21, 2011, each lasting 1–2 weeks. All vaccines were captured by the Kilifi Vaccine Monitoring System, a registry in which data clerks at 26 clinics serving the KHDSS linked vaccination at the point of delivery to the child's identification in the KHDSS.^9^

Children admitted to KCH (with the exception of patients with trauma or patients admitted for elective surgery) were investigated with a blood culture at the time of admission from 1999 to 2016.^10^ Adults (aged ≥15 years) admitted to KCH from 2007 to 2016 were investigated with a blood culture at the time of admission if there were signs or symptoms of invasive bacterial disease (eg, history of fever, axillary temperature <36·0°C or >37·5°C, signs of focal sepsis).^11^ Blood was cultured by use of an automated BACTEC instrument (BD Diagnostics, USA). From 1999 to 2016, apart from a brief change in practice in 2004–05 (appendix),^8^ the clinical indications for lumbar puncture were impaired consciousness or meningism in children younger than 5 years, prostration in children younger than 3 years, seizures (other than febrile seizures) in children younger than 2 years and suspicion of sepsis in children younger than 60 days, or suspected meningoencephalitis in adults. Cerebrospinal fluid (CSF) was cultured on horse blood and chocolate agar. Admitted patients were tested for HIV with two rapid antibody tests according to the Kenya national policy.^12^ Patients were treated according to Kenyan Ministry of Health or WHO guidelines.

Nasopharyngeal carriage of pneumococci was assessed through annual cross-sectional surveys of approximately 500 KHDSS residents of all ages selected at random from the KHDSS population register each year from 2009 to 2016. The methods are described elsewhere, with the exception that flocked swabs (Copan Diagnostics, USA) replaced rayon swabs in 2016.^13^

Isolates of *Streptococcus pneumoniae* from sterile-site and nasopharyngeal swab cultures were identified by optochin susceptibility; serotyping was performed by latex agglutination and Quellung reaction. If pneumococcal colonies of varying appearance were observed, only those of the dominant colony morphology were serotyped. Serogroup 6 isolates were tested by PCR for confirmation of serotype. Invasive isolates from 1999 to 2008 underwent repeat confirmatory serotyping by Quellung and multiplex PCR.^14^ Invasive isolates from 2008 to 2016 underwent real-time confirmatory serotyping by PCR; discordant results were resolved by a second PCR. A case of IPD was defined as isolation of *S pneumoniae* from a sterile site culture in an individual admitted to KCH who was resident in the KHDSS. VT isolates were those belonging to PCV10 serotypes (1, 4, 5, 6B, 7F, 9V, 14, 18C, 19F, and 23F). All other serotypes were classified as non-VT. Pneumococcal meningitis was defined as isolation of *S pneumoniae* from CSF or isolation of *S pneumoniae* from blood, accompanied by a CSF white blood cell count of at least 50 × 10^6^ cells per L or a ratio of CSF glucose to plasma glucose of less than 0·1.^15^ Pneumococcal pneumonia was defined as a case of IPD in a child with cough or difficulty breathing, and at least one of the following: lower chest wall indrawing, central cyanosis, inability to drink, convulsions, lethargy, prostration, or head nodding.^16^

### Statistical analysis

We designated Jan 1, 1999 through Dec 31, 2010, as the prevaccine era and Jan 1, 2012 through Dec 31, 2016, as the postvaccine era. The year of vaccine introduction, 2011, was excluded from the analysis of PCV10 effect. We calculated the age-stratified incidence of IPD in each year as the annual number of cases divided by the mid-year population in the KHDSS. We excluded admissions and person-years of observation during health-care worker strikes (appendix). Unadjusted incidence rate ratios (IRRs) were calculated for the postvaccine era compared with the prevaccine era by age group by means of negative binomial regression because of over-dispersion in the data. Possible confounders of the association between IPD and vaccine introduction included time (year), annual incidence of admissions, malaria admissions (ie, presence of malaria parasites by microscopy), moderate or severe malnutrition admissions (among children aged <5 years; defined as weight-for-age lower than −2 Z scores below the median of the WHO child growth standards),^17^ and compliance with recommendations for investigation by blood culture. Potential confounders with a p value of less than 0·1 in univariate analysis were included in the multivariable analysis; we used backward stepwise regression and excluded variables with a likelihood ratio test p value of at least 0·05. We built age-group-specific models for IPD caused by any serotype and applied the same structure within age group for VT and non-VT IPD. The percentage reduction in disease was calculated as 1 minus the adjusted IRR.

We calculated pneumococcal carriage prevalence ratios comparing nasopharyngeal carriage in the prevaccine and postvaccine eras as previously described.^13^ Briefly, prevalence ratios were modelled by means of log-binomial regression; if the models failed to converge we used Poisson regression with robust CIs. Adjusted prevalence ratios were age standardised to reflect the stratified sampling scheme by use of the inverse of the sampling ratio as population weights.

The significance of vaccine effect on serotype-specific IPD or carriage was tested with a Bonferroni correction (ie, for 25 serotypes, the correction was 0·05/25).

STATA 14.0 (Stata Corp, USA) was used for the analysis.

### Role of the funding source

The study was funded by Gavi, The Vaccine Alliance and The Wellcome Trust. The funders had no role in the study design, data analysis, data collection, data interpretation or writing of the report. The corresponding author had full access to all the data in the study and had final responsibility for the decision to submit the paper for publication.

## Results

**Figure 1:**
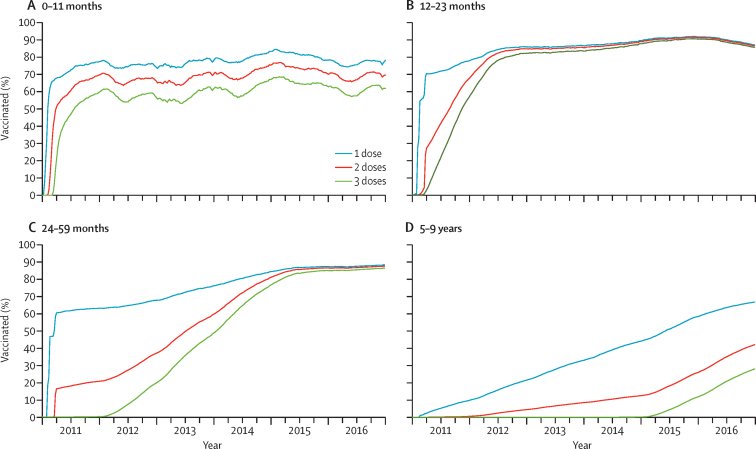
Proportion of children vaccinated with PCV10 (A) 0–11 months of age. (B) 12–23 months of age. (C) 24–59 months of age. (D) 5–9 years of age in the Kilifi Health and Demographic Surveillance System, 2011–16. PCV10=ten-valent pneumococcal conjugate vaccine.

Coverage with PCV10 increased sharply during the catch-up campaign and slowly thereafter (figure 1; appendix). Coverage with at least two PCV10 doses in 2–11-month- old infants was 80% by the end of 2011 and 84% by the end of 2016; coverage with at least one dose in 12–59-month-old infants was 66% by the end of 2011 and 87% by the end of 2016.

During the 18-year surveillance period, we identified 667 cases of IPD in 3 211 403 person-years of observation among KHDSS residents (appendix). The proportion of IPD cases detected by culture of blood or CSF and the proportion with HIV infection did not change between the prevaccine and postvaccine eras (table 1). Among children younger than 5 years, the median age of IPD cases was 14 months (IQR 7–30) in the prevaccine era and 20 months (IQR 6–38) in the PCV10 era. Throughout the 18-year surveillance period, several indicators suggested large improvements in the overall health of the population (appendix).

**Table 1:** Invasive pneumococcal disease among children and adults in the KHDSS admitted to the Kilifi County Hospital, in the prevaccine and postvaccine eras

		**Prevaccine era (1999–2010) n (%)**	**Postvaccine era (2012–16) n (%)**	p value*
**Age <5 years**				
Total admissions		31 422	6323	..
Mean annual admissions		2618	1264	<0·001
Recommended for blood culture		30 098 (96%)	5691 (90%)	<0·001
Blood culture collected†		29 234 (97%)	5353 (94%)	<0·001
Culture-confirmed IPD		401	34	..
	Age <24 months	273 (68%)	20 (59%)	0·27
	Male	229 (57%)	23 (68%)	0·23
	Cultured from blood specimen	390 (97%)	34 (100%)	0·33
	Cultured from CSF	71 (18%)	3 (9%)	0·19
	Pneumococcal meningitis‡	78 (19%)	3 (9%)	0·13
	HIV-infected§	25 (20%)	4 (15%)	0·55
	Died during the episode	70 (17%)	11 (32%)	0·03
**Age 5–14 years**				
Total admissions		5296	2060	..
Mean annual admissions		441	412	0·61
Recommended for blood culture		4483 (85%)	1592 (77%)	<0·001
Blood culture collected†		4200 (94%)	1438 (90%)	<0·001
Culture-confirmed IPD		127	22	..
	Male	66 (52%)	10 (45%)	0·57
	Cultured from blood specimen	121 (95%)	21 (95%)	0·97
	Cultured from CSF	31 (24%)	2 (9%)	0·11
	Pneumococcal meningitis‡	37 (29%)	4 (18%)	0·29
	HIV-infected§	7 (23%)	5 (36%)	0·39
	Died during the episode	30 (24%)	2 (9%)	0·13
**Age ≥15 years**				
Total admissions		5332	5163	..
Mean annual admissions		1326	1032	0·10
Recommended for blood culture		5048 (95%)	4597 (89%)	<0·001
Blood culture collected†		2107 (42%)	1875 (41%)	0·34
Culture-confirmed IPD		30	26	..
	Male	12 (40%)	11 (42%)	0·86
	Cultured from blood specimen	29 (97%)	23 (88%)	0·23
	HIV-infected§	4 (33%)	14 (64%)	0·09
	Died during the episode	12 (40%)	12 (46%)	0·64

KHDSS=Kilifi Health and Demographic Surveillance System. IPD=invasive pneumococcal disease. CSF=cerebrospinal fluid.

* p value computed by *t* test for comparison of means and χ^2^ for comparison of percentages.

† Among those recommended for blood culture.

‡ Defined as isolation of *Streptococcus pneumoniae* from CSF or isolation of *S pneumoniae* from blood, accompanied by a CSF white blood cell count of ≥50 × 10^6^ cells per L or greater, or a ratio of CSF glucose to plasma glucose <0·1.

§ Among those with HIV status ascertained.^15^

**Figure 2:**
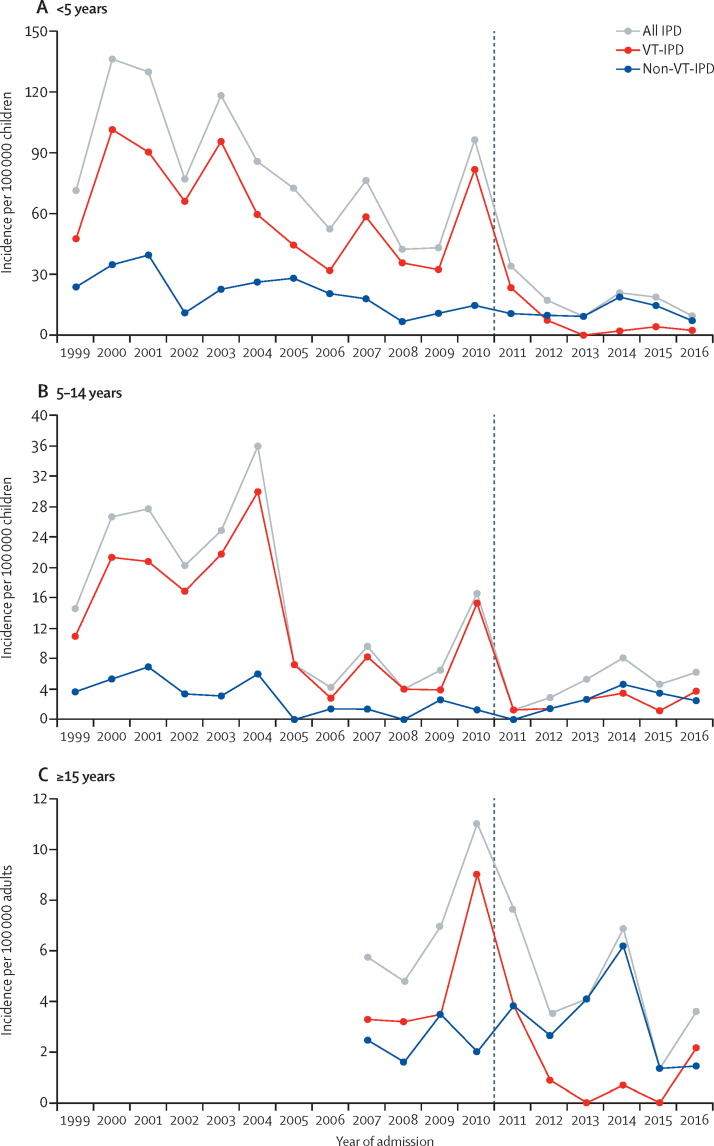
Incidence of overall, vaccine-type, and non-vaccine-type invasive pneumococcal disease in the Kilifi Health and Demographic Surveillance System, 1999–2016 (A) In individuals aged <5 years. (B) 5–14 years. (C) ≥15 years. Vertical dashed line indicates pneumococcal conjugate vaccine introduction. IPD=invasive pneumococcal disease. VT=vaccine serotype.

Among children younger than 5 years, the annual incidence of VT-IPD declined from 60·8 per 100 000 in the prevaccine era to 3·2 per 100 000 in the postvaccine era (table 2; figure 2; appendix) representing a reduction of 92% (95% CI 78–97; adjusted for year). The average annual number of VT-IPD cases fell from 25 (IQR 16–33) in the prevaccine era to 1 (IQR 1–2) in the postvaccine era. Seven children had VT-IPD in the postvaccine era: two were unvaccinated and five were age-appropriately vaccinated (appendix). Of the five children who developed VT-IPD after receipt of PCV10, two were noted to have malnutrition. A decline in incidence was observed for all PCV10 serotypes, and this was significant for serotypes 1 and 14, the most common serotypes in the prevaccine era. Serotype 1, in addition to causing a steady background of IPD, also caused occasional epidemics of IPD in Kilifi—for example, in 2010. Introduction of PCV10 effectively terminated this epidemic and resulted in the near-elimination of serotype 1 IPD (appendix). The incidence of non-VT IPD did not increase following introduction of PCV10 (IRR 1·31; 95% CI 0·65–2·64; table 2). To assess whether classification of serotype 6A as a non-VT masked our ability to detect an increase in non-VT IPD, we did a sensitivity analysis in which 6A was excluded from the non-VT group; results were similar (IRR 1·46; 95% CI 0·62–3·41). Overall in children younger than 5 years, use of PCV10 led to a 68% (95% CI 40–83) reduction in the incidence of all-serotype IPD, and an 85% (95% CI 66–93) reduction in the incidence of bacteraemic pneumococcal pneumonia.

**Table 2:** Incidence of IPD among children and adults in the KHDSS, in the prevaccine and postvaccine eras

	Prevaccine era (1999–2010)*		**Postvaccine era (2012–16)**		**Postvaccine *vs* prevaccine era**	
	n	Incidence per 100 000 (95% CI)	n	Incidence per 100 000 (95% CI)	IRR† (95% CI)	Adjusted IRR‡ (95% CI)
**IPD caused by any serotype**						
<2 months	43	240·2 (173·9–323·6)	1	7·9 (0·2–44·0)	0·03 (0·00–0·25)	0·13 (0·01–1·24)
<5 years	401	81·6 (73·8–89·9)	34	15·3 (10·6– 21·40)	0·18 (0·11–0·29)	0·32 (0·17–0·60)
5–14 years	127	15·8 (13·2–18·8)	22	5·5 (3·5–8·4)	0·34 (0·17–0·67)	0·47 (0·24–0·92)
≥15 years	30	6·9 (4·7–9·9)	26	3·9 (2·5–5·7)	0·56 (0·33–0·96)	0·63 (0·36–1·08)
**IPD caused by serotypes in PCV10**§						
<2 months	31	173·2 (117·7–245·8)	0	0·0	Not estimable	..
<5 years	299	60·8 (54·1–68·1)	7	3·2 (1·3–6·5)	0·05 (0·02–0·12)	0·08 (0·03–0·22)
5–14 years	105	13·1 (10·7–15·8)	10	2·5 (1·2–4·6)	0·19 (0·08–0·43)	0·26 (0·11–0·59)
≥15 years	20	4·6 (2·8–7·1)	5	0·7 (0·2–1·7)	0·16 (0·06–0·45)	0·19 (0·07–0·51)
**IPD caused by serotypes not in PCV10**						
<2 months	12	67·0 (34·3–17·0)	1	7·9 (0·2–4·0)	0·12 (0·02–0·94)	0·26 (0·02–3·45)
<5 years	102	20·8 (16·9–25·2)	27	12·2 (8·0–17·7)	0·58 (0·35–0·95)	1·31 (0·65–2·64)
5–14 years	22	2·7 (1·7–4·2)	12	3·0 (1·6–5·3)	1·10 (0·54–2·22)	1·45 (0·66–3·20)
≥15 years	10	2·3 (1·1–4·2)	21	3·1 (1·9–4·8)	1·36 (0·62–2·98)	1·47 (0·67–3·21)
**Bacteraemic pneumococcal pneumonia caused by any serotype**						
<5 years	212	43·1 (37·5–49·3)	19	8·6 (5·2–13·4)	0·20 (0·11–0·35)	0·15 (0·07–0·34)
5–14 years	57	7·1 (5·4–9·2)	11	2·8 (1·4–5·0)	0·38 (0·17–0·86)	0·49 (0·21–1·11)
**Pneumococcal meningitis caused by any serotype**						
<5 years	78	15·9 (12·5–19·8)	3	1·4 (0·3–4·0)	0·08 (0·02–0·29)	0·31 (0·08–1·21)
5–14 years	37	4·6 (3·2–6·4)	4	1·0 (0·3–2·6)	0·21 (0·06–0·71)	0·31 (0·09–1·00)

IPD=invasive pneumococcal disease. KHDSS=Kilifi Health and Demographic Surveillance System. IRR=incidence rate ratio. PCV10=pneumococcal conjugate vaccine.

* For individuals ≥15 years, the prevaccine era was 2007–10.

† IRR estimated using negative binomial regression.

‡ IRR estimated using negative binomial regression, adjusted for confounding factors significant in the age-specific all-type IPD models: year (age groups <2 months and <5 years), blood culture collection (age groups 5–14 years and ≥15 years).

§ PCV serotypes=serotypes 1, 4, 5, 6B, 7F, 9V, 14, 18C, 19F, and 23F.

Among children too young to be vaccinated (ie, <2 months of age), the incidence of VT-IPD declined from 173·2 per 100 000 in the prevaccine era to 0·0 per 100 000 in the postvaccine era. A significant decline in the incidence of VT-IPD was also observed among individuals aged 5–14 years (74% reduction, 95% CI 41–89; adjusted for blood culture ascertainment), and among those aged 15 years or more (81% reduction, 95% CI 49–93; adjusted for blood culture ascertainment; figure 2; appendix).

Overall 4066 KHDSS residents were enrolled in the nasopharyngeal carriage surveys (appendix). VT carriage declined among individuals younger than 5 years (with significant reductions for serotypes 6B, 19F, and 23F), 5–14 years, and aged 15 years or more. Non-VT carriage increased significantly in in all age groups (table 3; appendix). Among children aged less than 5 years, carriage of vaccine-related serotype 19A increased; no effect on carriage of serotype 6A was observed. In 2016, carriage of VT pneumococci was detected in ten (6%) of 156 children aged less than 5 years, all of whom had received three doses of PCV10, and seven (7%) of 99 children aged 5–14 years, none of whom had received PCV10.

**Table 3:** Carriage prevalence and prevalence ratios for nasopharyngeal carriage of *Streptococcus pneumoniae* in the prevaccine and postvaccine eras

	**Carriage prevalence prevaccine era (2009–10) n (%)**	**Carriage prevalence postvaccine era (2012–16) n (%)**	**Crude prevalence ratio (95% CI)**	Age-standardised adjusted prevalence ratio (95% CI)*
**All *S pneumoniae***				
<5 years	229 (74·4%)	606 (76·0%)	1·02 (0·95–1·10)	1·00 (0·92–1·08)
5–14 years	103 (52·6%)	237 (48·5%)	0·92 (0·78–1·08)	0·96 (0·81–1·13)
≥15 years	123 (24·0%)	287 (22·8%)	0·95 (0·79–1·14)	1·00 (0·81–1·22)
**Vaccine-type *S pneumoniae***				
<5 years	104 (33·8%)	70 (8·8%)	0·26 (0·20–0·34)	0·26 (0·19–0·35)
5–14 years	30 (15·3%)	29 (5·9%)	0·39 (0·24–0·63)	0·38 (0·22–0·64)
≥15 years	29 (5·7%)	17 (1·4%)	0·24 (0·13–0·43)	0·23 (0·12–0·44)
**Non-vaccine-type *S pneumoniae***				
<5 years	125 (40·6%)	557 (69·9%)	1·72 (1·49–1·99)	1·71 (1·47–1·99)
5–14 years	73 (37·2%)	216 (44·2%)	1·19 (0·96–1·46)	1·25 (1·01–1·54)
≥15 years	94 (18·3%)	286 (22·7%)	1·24 (1·01–1·53)	1·30 (1·05–1·63)

* Models adjusted for number of children aged <10 years in household, month of swab collection, cough or rhinorrhea in preceding 14 days (all age groups); antibiotic use in the preceding 14 days also remained in the model for children aged 5–14 years.

In an exploratory post-hoc analysis using negative binomial regression, adjusting for calendar-year, the introduction of PCV10 was associated with a non-significant reduction in invasive *S aureus* disease in children aged less than 5 years (IRR 0·65; 95% CI 0·36–1·18).

## Discussion

Using a longstanding, integrated clinical, laboratory, and demographic surveillance system, we documented a 92% reduction in VT-IPD in children younger than 5 years and substantial indirect protection in Kilifi, Kenya following introduction of PCV10 to the routine infant immunisation schedule, accompanied by a catch-up campaign. Kenya was the first African country to include PCV10 in its routine childhood immunisation programme. This study provides the first population-level evidence of a direct and indirect effect of a PCV10 programme in a low-income country and does not find significant evidence of serotype replacement disease in the first 6 years of PCV10 use.

Following introduction of PCVs, a large decline in IPD was documented in numerous developed world settings; however, serotype distributions in carriage and IPD differ by geographical area and there have been few opportunities to examine PCV10 effect in a developing country.^18^ In Kilifi, we observed a 68% reduction in IPD caused by any serotype and a 92% reduction in VT-IPD among children younger than 5 years, consistent with findings in middle-income and high-income settings in which PCV10 or PCV13 were used. 4 years after PCV13 introduction, IPD was reduced by 64% in US children younger than 5 years and 46% in British children younger than 2 years.^19^, ^20^ Within the first 3–5 years of PCV13 use, the incidence of IPD caused by the six serotypes present in PCV13 but not PCV7 declined 93% in US children younger than 5 years, 80% in Alaska Native children younger than 5 years, 89% in British children younger than 2 years, and 82% in Gambian children aged 2–23 months.^19^, ^20^, ^21^, ^22^ In Latin American countries using PCV10 or PCV13, effectiveness against VT-IPD has been estimated at 56%–84%.^23^ Contributing to the observed reduction in IPD in Kilifi were an 85% reduction in bacteraemic pneumococcal pneumonia incidence and a 69% reduction in pneumococcal meningitis incidence in children younger than 5 years. PCV effect on these important clinical outcomes has been documented elsewhere.^24^, ^25^, ^26^ Given that the majority of pneumococcal disease is comprised of pneumonia, it is notable that PCV10 introduction in Kenya was associated with a 27% reduction in childhood hospital admissions with clinically-defined pneumonia and a 48% reduction in childhood hospital admissions with radiologically confirmed pneumonia.^27^ Although the introduction of PCV10 reduced the overall incidence of IPD by 68% in children aged less than 5 years, almost a third of serious pneumococcal disease remains. Higher valency conjugate vaccines or serotype-independent vaccines, which are in development, are likely to lead to greater reductions in pneumococcal disease.

In the prevaccine era, IPD was driven by epidemics of serotypes 1 and 5 in Kilifi. PCV10 use not only reduced the incidence of disease but obliterated IPD epidemics, as was also observed in the USA.^28^ We did not observe a reduction in IPD caused by vaccine-related serotypes 6A or 19A; this is consistent with the findings from the nasopharyngeal carriage surveys in Kilifi. By contrast, an analysis of the long-term effect of PCV10 in Finland, administered as two primary doses in infancy followed by a booster dose (ie, 2 plus 1), documented a reduction in IPD caused by serotype 6A but not 19A.^29^ A case-control analysis in Brazil, which used a 3 plus 1 schedule at the time, documented PCV10 effectiveness against 19A but not 6A.^30^ Data suggest that a booster dose might achieve greater protection against vaccine-related serotypes but additional studies are required to quantify this.^31^

Notably, we observed protection among infants too young to be vaccinated and among older children and adults. In South Africa a decline in VT-IPD was noted among adults aged 25–44 years within 4 years of PCV7 introduction.^32^ However, indirect effects were not observed within the 3 years following introduction of PCV13 in The Gambia.^22^ A catch-up campaign was not done in The Gambia but in Kilifi this probably accelerated population protection.^33^ The full magnitude of PCV10 effect might take longer to achieve in the absence of a catch-up campaign.

The indirect protection afforded by PCVs is driven by the reduction in nasopharyngeal carriage of VT pneumococci among vaccinated children. In Kilifi, there was a significant reduction in carriage of VT pneumococci in both vaccinated and unvaccinated populations within 6 months of PCV10 introduction.^13^ However, although vaccine-type carriage has declined, VT pneumococci continue to be identified in 6% of children aged less than 5 years and 8% of infants in Kilifi, compared with less than 1–2% in other countries that use PCVs.^34^, ^35^, ^36^, ^37^, ^38^ Residual VT carriage has also been documented in other parts of Kenya. Among healthy children aged less than 5 years, the prevalence of VT carriage ranged between 10% and 11% in parts of western Kenya that used a catch-up campaign and between 4% and 14% in communities around Nairobi without catch-up; the difference between areas with and without catch-up could not be distinguished statistically.^39^, ^40^ The persistence of VT carriage might reflect a higher force of infection in Kenya and it indicates continued risk for VT-IPD in unvaccinated or under-vaccinated children, and adults.^41^ Another possible explanation for persistent VT carriage is that, unlike most middle-income and high-income countries, Kenya introduced PCV10 without a booster dose in the second year of life. Many low-income countries have introduced PCV with three primary doses without a booster (3 plus 0 schedule), and it will be important to establish whether the absence of a booster dose, which might lead to more rapid waning of immunity, leads to a persistent transmission reservoir, vaccination failures, or rebound disease incidence. An analysis of the PCV vaccination programme in Australia suggested waning effectiveness, particularly beyond 24 months after the third dose, and suboptimal community protection with a 3 plus 0 schedule.^42^ These findings led the Australian National Immunisation Technical Advisory Group to recommend a change in the PCV schedule to include a booster dose.^43^

In addition to persistent VT carriage, we also observed a 71% increase in carriage of non-VT pneumococci (particularly serotype 19A) in children younger than 5 years. The PCV-associated decline in VT carriage and corresponding increase in non-VT carriage has been well described. Although an increase in non-VT IPD disease has been reported in settings that use expanded valency PCVs, the increases have generally been small compared with the decline in VT-IPD.^19^, ^20^ A significant increase in non-VT IPD in adults has offset the benefits of PCV use in some settings such as the UK and Brazil; however, enhanced surveillance in the postvaccine period might account for some of this increase in Brazil.^44^, ^45^ Although our surveillance for non-VT IPD did not detect a significant increase in any age group, the direction of change was positive in all age groups 2 months and above (IRRs 1·31–1·47). Given the low baseline incidence of non-VT IPD, comprising only one quarter of the prevaccine burden of IPD among children aged less than 5 years, the power of the study was only sufficient (ie, >80%) to detect at least a 2·3-fold change (appendix). The small relative increase that was observed did not translate into a significant absolute rise in incidence. To clarify these emerging trends, it will be important to continue to monitor children and adults for pneumococcal disease in the existing surveillance setting for several years to come. Not only is this essential to detect possible emergence of non-VT disease, but also to monitor for rebound VT disease that might occur if the indirect effects achieved by the catch-up campaign wane in the absence of booster dose to sustain immunity and suppress circulation of VT pneumococci.

The before–after study design, which is the principal method for evaluation of the population effect of vaccines, has inherent weaknesses. In Kilifi, general health improved slowly over the surveillance period. The incidence of hospital admissions for various illnesses declined. The reduction in non-VT IPD over time among infants aged less than 2 months, by contrast with the rise in older age groups, suggests specific improvements in maternity services and infant care. The incidence of HIV in the population has not been systematically measured and was not included in the analysis; however, the prevalence of HIV among women seeking antenatal care was less than 5% over the surveillance period and it is therefore unlikely that changes in HIV incidence would have significantly confounded estimates of vaccine effectiveness. Several factors argue that the observed reduction in IPD in Kilifi is attributable to the introduction of PCV10, although the exact estimate of effect might be subject to some residual confounding in the variables mentioned above. Consistent surveillance methods were used over a long period of time and changes in VT-IPD occurred abruptly at the same time as vaccine introduction with a catch-up campaign, and simultaneously with marked changes in VT carriage prevalence. Systematically collected data on a wide range of possible confounders were included in the analysis.

Pneumococcal disease remains a leading vaccine-preventable cause of childhood mortality, and most of these deaths occur in Africa. To date, 141 countries, including 58 Gavi-eligible countries, have introduced a PCV into their national childhood immunisation programmes.^3^ 16 countries are in the process of transitioning out of Gavi support and five have reached the end of Gavi support. The PCV programme is the most expensive component of the national immunisation schedule and the sustainability of PCV vaccination in low-income countries will depend on the demonstrable effect of PCV in reducing childhood morbidity and mortality. Because of the necessity for stable prevaccine surveillance, evaluations of PCV effect are rare in Africa. On the basis of this carefully standardised, prospectively designed, 18-year surveillance study, we conclude that use of PCV10 in tropical Africa will lead to substantial health benefits for the whole population. Continued surveillance will elucidate the long-term sustainability of these benefits.
