# Supplementary material for: Effect of ten-valent pneumococcal conjugate vaccine on invasive pneumococcal disease and nasopharyngeal carriage in Kenya: a longitudinal surveillance study
Source: Lancet. 2019 May 25;393(10186):2146–54. doi: 10.1016/S0140-6736(18)33005-8 (PMC6548991; doi:10.1016/S0140-6736(18)33005-8)
Supplement: Supplementary appendix [file mmc1.pdf]

THE LANCET

Supplementary appendix

This appendix formed part of the original submission and has been peer reviewed. We post it as supplied by the authors.

Supplement to: Hammitt LL, Etyang AO, Morpeth SC, et al. Effect of ten-valent pneumococcal conjugate vaccine on invasive pneumococcal disease and nasopharyngeal carriage in Kenya: a longitudinal surveillance study. *Lancet* 2019; published online April 15. http://dx.doi.org/10.1016/S0140-6736(18)33005-8.

Supplementary webappendix

Effect of ten-valent pneumococcal conjugate vaccine on invasive pneumococcal disease and nasopharyngeal carriage in Kenya: a longitudinal surveillance study

Supplemental Table 1. Coverage* with PCV10 among children in the Kilifi Health and Demographic Surveillance System, 2011-2016

Vaccinated with PCV10	2011	2012	2013	2014	2015	2016
2-11 months (≥2 doses)	79·9%	76·4%	81·4%	87·7%	84·2%	84·3%
12-23 months (≥1 dose)	76·0%	84·4%	85·6%	89·3%	91·6%	86·9%
24-59 months (≥1 dose)	62·7%	66·9%	74·6%	82·8%	86·5%	87·6%
60-143 months (≥1 dose)	7·2%	15·7%	24·1%	32·4%	42·8%	53·8%

*Cross-sectional prevalence of PCV10 coverage at the end of each calendar year.

Supplemental Table 2. Dates of health care worker strikes at Kilifi County Hospital, 1999-2016

Dates	Number of days	Ward(s) affected
1 Sept 2009 – 31 Mar 2010	212*	Adult
5 Dec 2011 - 12 Dec 2011	8	Adult and paediatric
1 Mar 2012 - 15 Mar 2012	15	Adult and paediatric
13 Sept 2012 - 4 Nov 2012	22	Adult and paediatric
2 Dec 2012 - 22 Dec 2012	21	Adult and paediatric
23 Dec 2012 - 13 Jan 2013	22	Adult
16 Jan 2013 - 11 Feb 2013	27	Adult and paediatric
10 Dec 2013 - 21 Dec 2013	12	Adult and paediatric
5 Dec 2016 - 31 Dec 2016	27	Adult and paediatric

*During 1 Sept 2009–31 Mar 2010, health care workers withdrew from participation in the surveillance system although the hospital wards remained open. During the other strike periods, the hospital was effectively closed.

Supplemental Table 3. Invasive pneumococcal disease among children and adults in the KHDSS admitted to the Kilifi County Hospital, 1999-2016

	**1999** n (%)	**2000** n (%)	**2001** n (%)	**2002** n (%)	**2003** n (%)	**2004** n (%)	**2005** n (%)	**2006** n (%)	**2007** n (%)	**2008** n (%)	**2009** n (%)	**2010** n (%)	**2011** n (%)	**2012** n (%)	**2013** n (%)	**2014** n (%)	**2015** n (%)	**2016** n (%)
**Age <5 years**																		
Admissions	3,568	3,590	3,513	2,378	2,769	2,705	2,305	2,454	2,126	1,982	2,131	1,901	1,617	1,224	971	1,467	1,440	1,221
Eligible for blood culture	3541 (99)	3526 (98)	3446 (98)	2314 (97)	2671 (96)	2573 (95)	215 (93)5	2324 (95)	1964 (92)	1819 (92)	2005 (94)	1760 (93)	1512 (94)	1098 (90)	854 (88)	1348 (92)	1292 (90)	1099 (90)
Eligible and blood culture done	3490 (99)	3482 (99)	3394 (98)	2286 (99)	2634 (99)	2524 (98)	2109 (98)	2269 (98)	1832 (93)	1687 (93)	1879 (94)	1648 (94)	1438 (95)	1017 (93)	805 (94)	1274 (95)	1228 (95)	1029 (94)
Lumbar puncture done	712 (20)	884 (25)	796 (23)	616 (26)	542 (20)	311 (11)	443 (19)	658 (27)	565 (27)	504 (25)	509 (24)	490 (26)	398 (25)	316 (26)	244 (25)	365 (25)	334 (23)	264 (22)
Culture-confirmed IPD																		
No. with all serotypes	24	47	46	28	47	36	31	23	34	19	20	46	16	7	4	10	9	4
No. with VT IPD	16 (67)	35 (74)	32 (70)	24 (86)	38 (81)	25 (69)	19 (61)	14 (61)	26 (76)	16 (84)	15 (75)	39 (85)	11 (69)	3(43)	0	1 (10)	2 (22)	1(25)
No. with VT *S. pn.* cultured in CSF	4 (17)	5 (11)	8 (17)	8 (29)	8 (17)	3 (8)	2 (6)	1 (4)	7 (21)	1 (5)	0	3 (7)	0	0	0	0	0	0
No. with *S. pn.* meningitis	8 (33)	10 (21)	10 (22)	10 (36)	8 (17)	5 (14)	6 (19)	2 (9)	10 (29)	3 (16)	1 (5)	5 (11)	1 (6)	1 (14)	0	0	2 (22)	0
No. with non-VT IPD	8 (33)	12 (26)	14 (30)	4 (14)	9 (19)	11 (31)	12 (39)	9 (39)	8 (24)	3 (16)	5 (25)	7 (15)	5 (31)	4 (57)	4 (100)	9 (90)	7 (78)	3 (75)
Age <24 months	18 (75)	36 (77)	32 (70)	22 (79)	27 (57)	24 (67)	26 (84)	17 (74)	24 (71)	12 (63)	15 (75)	20 (43)	8 (50)	5 (71)	2 (50)	5 (50)	6 (67)	2 (50)
Male	15 (63)	27 (57)	26 (57)	20 (71)	27 (57)	21 (58)	17 (55)	11 (48)	17 (50)	14 (74)	13 (65)	21 (46)	9 (56)	6 (86)	3 (75)	6 (60)	6 (67)	2 (50)
Died during the episode	9 (38)	2 (4)	13 (28)	8 (29)	9 (19)	9 (25)	4 (13)	2 (9)	7 (21)	2 (11)	2 (10)	3 (7)	1 (6)	3 (43)	1 (25)	2 (20)	5 (56)	0
**Age 5-14 Years**																		
Admissions	531	517	494	341	430	382	429	426	423	412	439	472	439	292	308	528	527	405
Eligible for blood culture	523 (98)	504 (97)	479 (97)	302 (89)	380 (88)	297 (78)	331 (77)	322 (76)	331 (78)	317 (77)	322 (73)	375 (79)	328 (75)	220 (75)	236 (77)	433 (82)	420 (80)	283 (70)
Eligible and blood culture done	504 (96)	482 (96)	447 (93)	294 (97)	372 (98)	285 (96)	319 (96)	307 (95)	294 (89)	257 (81)	290 (90)	349 (93)	303 (92)	204 (93)	213 (90)	404 (93)	380 (90)	237 (84)
Lumbar puncture done	79 (15)	74 (14)	61 (12)	42 (12)	50 (12)	39 (10)	33 (8)	54 (13)	46 (11)	48 (12)	46 (10)	87 (18)	60 (14)	59 (20)	75 (24)	92 (17)	73 (14)	36 (9)
Culture-confirmed IPD																		
No. with all serotypes	8	15	16	12	16	24	5	3	7	3	5	13	1	2	4	7	4	5
No. with VT IPD	6 (75)	12 (80)	12 (75)	10 (83)	14 (88)	20 (83)	5 (100)	2 (67)	6 (86)	3 (100)	3 (60)	12 (92)	1 (100)	1 (50)	2 (50)	3 (43)	1 (25)	3 (60)
No. with VT *S. pn.* cultured in CSF	3 (38)	2 (13)	3 (19)	4 (33)	4 (25)	4 (17)	0	1 (33)	2 (29)	1 (33)	0	1 (8)	0	0	1 (25)	0	0	0
No. with *S. pn.* meningitis	5 (63)	3 (20)	6 (38)	4 (33)	5 (31)	6 (25)	0	1 (33)	3 (43)	1 (33)	0	3 (23)	0	1 (50)	2 (50)	0	1 (25)	0
No. with non-VT IPD	2 (25)	3 (20)	4 (25)	2 (17)	2 (13)	4 (17)	0	1 (33)	1 (14)	0	2 (40)	1 (8)	0	1 (50)	2 (50)	4 (57)	3 (75)	2 (40)
Male	5(63)	7 (47)	5 (31)	5 (42)	10 (63)	14 (58)	4 (80)	2 (67)	3 (43)	3 (100)	3 (60)	5 (38)	0	2 (100)	3 (75)	2 (29)	2 (50)	1 (20)
Died during the episode	3 (2)	4 (27)	1 (6)	2 (17)	5 (31)	4 (17)	2 (40)	0	2 (29)	2 (67)	2 (40)	3 (23)	0	0	0	2 (29)	0	0
**Age ≥15 years**																		
Admissions									1,201	1,673	1,252	1,181	1,080	758	834	1,061	1,190	1,320
Eligible for blood culture									1149 (96)	1607 (96)	1172 (94)	1097 (93)	976 (90)	678 (89)	755 (91)	956 (90)	1050(88)	1158 (88)
Eligible and blood culture done									454 (40)	518 (32)	549 (47)	563 (51)	392 (40)	283 (42)	284 (38)	428 (45)	397 (38)	483 (42)
Culture-confirmed IPD																		
No. with all serotypes									7	6	6	11	10	4	5	10	2	5
No. with VT IPD									4 (57)	4 (67)	3 (50)	9 (82)	5 (50)	1 (25)	0	1 (10)	0	3 (60)
No. with VT *S. pn.* cultured in CSF									0	1 (17)	0	3 (27)	2 (20)	1 (25)	0	0	0	1 (20)
No. with *S. pn.* meningitis									0	1 (17)	0	3 (27)	3 (30)	2 (50)	2 (40)	2 (20)	0	2 (40)
No. with non-VT IPD									3 (43)	2 (33)	3 (50)	2 (18)	5 (50)	3 (75)	5 (100)	9 (90)	2 (100)	2 (40)
Male									3 (43)	2 (33)	2 (33)	5 (45)	4 (40)	3 (75)	1 (20)	3 (30)	1 (50)	3 (60)
Died during the episode									2 (29)	1 (17)	4 (67)	5 (45)	6 (60)	2 (50)	1 (20)	5 (50)	1 (50)	3 (60)

Abbreviations: IPD, invasive pneumococcal disease; VT, vaccine-type; *S. pn.*, *Streptococcus pneumoniae*; CSF, cerebrospinal fluid.

Supplemental Table 4. Incidence (per 100,000) of invasive pneumococcal disease by serotype category and age group in the Kilifi Health and Demographic Surveillance System, 1999-2016

Serotype category and age group	Pre-vaccine era (1999-2010)			2011			2012			2013			2014			2015			2016		
	n	Inc	95% CI	n	Inc	95% CI	n	Inc	95% CI	n	Inc	95% CI	n	Inc	95% CI	n	Inc	95% CI	n	Inc	95% CI
**All-type**																					
<2 mos	43	240·2	(173·9, 323·9)	1	37·7	(1·0, 210·3)	0			0			0			1	35·3	(0·9, 196·5)	0		
<5 yrs	401	81·6	(73·8, 89·9)	16	34·1	(19·5, 55·3)	7	17·2	(6·9, 35·4)	4	9·3	(2·5, 23·7)	10	20·9	(10·0, 38·5)	9	18·8	(8·6, 35·7)	4	9·5	(2·6, 24·4)
5-14 yrs	127	15·8	(13·2, 18·8)	1	1·3	(0·03, 7·1)	2	2·9	(0·4, 10·5)	4	5·3	(1·4, 13·6)	7	8·1	(3·3, 16·7)	4	4·6	(1·3, 11·9)	5	6·2	(2·0, 14·5)
≥15 yrs	30	2·4	(1·6, 3·4)	10	7·6	(3·7, 14·0)	4	3·5	(1·0, 9·0)	5	4·1	(1·3, 9·5)	10	6·9	(3·3, 12·6)	2	1·4	(0·2, 4·9)	5	3·6	(1·2, 8·4)
**PCV10-type**																					
<2 mos	31	173·2	(117·7, 245·8)	0			0			0			0			0			0		
<5 yrs	299	60·8	(54·1, 68·1)	11	23·4	(11·7, 41·9)	3	7·4	(1·5, 21·5)	0			1	2·1	(0·1, 11·7)	2	4·2	(0·5, 15·1)	1	2·4	(0·1, 13·3)
5-14 yrs	105	13·1	(10·7, 15·8)	1	1·3	(0·03, 7·1)	1	1·5	(0·04, 8·1)	2	2·7	(0·3, 9·6)	3	3·5	(0·7, 10·2)	1	1·2	(0·03, 6·5)	3	3·7	(0·8, 10·9)
≥15 yrs	20	1·6	(1·0, 2·4)	5	3·8	(1·2, 8·9)	1	0·9	(0·02, 4·9)	0			1	0·7	(0·02, 3·8)	0			3	2·2	(0·4, 6·3)
**Non-PCV10 type**																					
<2 mos	12	67·0	(34·3,117·1)	1	37·7	(1·0, 210·2)	0			0			0			1	35·3	(0·9, 196·5)	0		
<5 yrs	102	20·8	(16·9, 25·2)	5	10·7	(3·5, 24·9)	4	9·8	(2·7, 25·2)	4	9·3	(2·5, 23·7)	9	18·8	(8·6, 35·7)	7	14·6	(5·9, 30·1)	3	7·1	(1·5, 20·9)
5-14 yrs	22	2·7	(1·7, 4·2)	0			1	1·5		2	2·7		4	4·6	(1·3, 11·9)	3	3·5	(0·7, 10·2)	2	2·5	(0·3, 9·0)
≥15 yrs	10	0·8	(0·4, 1·5)	5	3·8	(1·2, 8·9)	3	2·6	(0·5, 7·7)	5	4·1	(1·3, 9·5)	9	6·2	(2·8, 11·7)	2	1·4	(0·2, 4·9)	2	1·4	(0·8, 5·2)

*For individuals ≥15 years, the pre-vaccine era was 2007-2010.

Abbreviations: Inc, Incidence per 100,000; CI, confidence interval; mos, months; yrs, years.

Supplemental Table 5. Serotype-specific incidence (per 100,000) of invasive pneumococcal disease among children aged <5 years in the Kilifi Health and Demographic Surveillance System in the pre-vaccine era (1999-2010) and post-vaccine era (2012-2016)

	Pre-vaccine era (1999-2010)			Post-vaccine era (2012-2016)*			Post- vs pre-vaccine era			
**Serotype**	**n**	**Incidence**	**95% CI**	**n**	**Incidence**	**95% CI**	**IRR†**	**95%CI**	**Adjusted IRR‡**	**95% CI**
**PCV10-type**										
1	104	21·2	(17·3, 25·6)	2	0·9	(0·1, 3·3)	0·04	(0·01, 0·2)	0·1	(0·01, 0·4)
4	7	1·4	(0·6, 2·9)	0	0		0		0	
5	35	7·1	(5·0, 9v9)	2	0·9	(0·1, 3·3)	0·1	(0·03, 0·6)	0·2	(0·02, 1·7)
6B	34	6·9	(4·8, 9·7)	0	0		0		0	
7F	3	0·6	(0·1, 1·8)	0	0		0		0	
9V	7	1·6	(0·7, 3·2)	0	0		0		0	
14	46	9·4	(6·9, 12·5)	1	0·5	(0·01, 2·5)	0·1	(0·01, 0·4)	0·1	(0·01, 0·9)
18C	16	3·3	(1·9, 5·3)	0	0		0		0	
19F	14	2·9	(1·6, 4·8)	2	0·9	(0·1, 3·3)	0·3	(0·1, 1·6)	0·4	(0·04, 3·6)
23F	33	6·7	(4·6, 9·4)	0	0		0		0	
**Non-PCV10 type§**										
6A	41	8·3	(6·0, 11·3)	5	2·3	(0·7, 5·3)	0·3	(0·1, 0·7)	0·8	(0·2, 3·1)
19A	11	2·2	(1·1, 4·0)	5	2·3	(0·7, 5·3)	1·0	(0·3, 3·6)	1·2	(0·1, 12·2)
3	8	1·6	(0·7, 3·2)	0			0		0	
12F	7	1·4	(0·6, 2·9)	2	0·9	(0·1, 3·3)	0·6	(0·1, 3·9)	2·5	(0·1, 75·5)
10A	6	1·2	(0·5, 2·7)	0			0		0	
35B	6	1·2	(0·5, 2·7)	0			0		0	
15A	3	0·6	(0·1, 1·8)	7	3·2	(1·8, 6·5)	5·2	(1·3, 20·0)	4·2	(0·2, 74·2)
34	3	0·6	(0·1, 1·8)	0			0		0	
13	2	0·4	(0·1, 1·5)	0			0		0	
23A	2	0·4	(0·1, 1·5)	0			0		0	
24F	2	0·4	(0·1, 1·5)	1	0·5	(0·01, 2·5)	1·1	(0·1, 12·2)	2·2	(0·1, 55·3)
38	2	0·4	(0·1, 1·5)	2	0·9	(0·1, 3·3)	2·2	(0·3, 15·7)	0·8	(0·02, 34·0)
16F	1	0·2	(0·0, 1·1)	2	0·9	(0·1, 3·3)	4·4	(0·4, 48·9)	32·6	(1·8, 578·6)

Abbreviations: CI, confidence interval; IRR, incidence rate ratio.
IRR estimates in bold indicate p<0·002 (corresponding to the adjustment for multiple comparisons).
*Vaccination status of PCV10-type cases in the post-vaccine era: All cases occurred in children who were fully vaccinated (either three primary doses or a single toddler dose) except for one case each of serotype 19F and serotype 14 that occurred in unvaccinated children.
†IRR estimated using negative binomial regression.
‡IRR estimated using negative binomial regression, adjusted for year.

§Restricted to serotypes detected in ≥2 cases in either the pre- or post-PCV10 period. Other serotypes detected included serotype 2 (n=1 pre-vaccine, n=0 post-vaccine), 7C (n=0 pre-vaccine, n=1 post-vaccine), 9L (n=1 pre-vaccine, n=0 post-vaccine), 15B (n=1 pre-vaccine, n=1 post-vaccine), 15C (n=1 pre-vaccine, n=0 post-vaccine), 18A (n=1 pre-vaccine, n=0 post-vaccine), 21 (n=1 pre-vaccine, n=0 post vaccine), 23B (n=1 pre-vaccine, n=1 post-vaccine), 33B (n=1 pre-vaccine, n=0 post-vaccine).

Supplemental Table 6. Characteristics of cases with PCV10-type invasive pneumococcal disease among children aged <5 years in the Kilifi Health and Demographic Surveillance System in the post-vaccine era (2012-2016)

Year of culture	Age (months)	Disease syndrome	Serotype	Underlying conditions	HIV infection status	PCV10 vaccination status
2012	27	Meningitis	19F	None noted	Negative	Unvaccinated
2012	7	Very severe pneumonia	19F	Moderate malnutrition	Negative	3 primary doses in infancy
2012	43	Meningitis	5	None noted	Negative	Single toddler dose at 27 months of age
2014	58	Very severe pneumonia	5	None noted	Unknown	Single toddler dose at 22 months of age
2015	9	Meningitis	1	Severe malnutrition	Negative	3 primary doses in infancy
2015	37	Meningitis	1	None noted	Negative	3 primary doses in infancy
2016	6	Very severe pneumonia	14	Severe malnutrition	Negative	Unvaccinated

Supplemental Table 7. Epidemiological characteristics of nasopharyngeal carriage study participants in the Kilifi Health and Demographic Surveillance System, 2009-2016

	2009 (n=506)	2010 (n=511)	2011 (n=504)	2012 (n=510)	2013 (n=503)	2014 (n=513)	2015 (n=510)	2016 (n=509)
Female	283 (56%)	275 (54%)	263 (52%)	277 (54%)	258 (51%)	302 (59%)	288 (57%)	291 (57%)
Age <5 years	152 (30%)	156 (31%)	151 (30%)	164 (32%)	162 (32%)	160 (31%)	154 (30%)	156 (31%)
Age 5-14 years	97 (19%)	99 (19%)	97 (19%)	94 (18%)	94 (19%)	100 (20%)	102 (20%)	99 (20%)
Age ≥15 years	257 (51%)	256 (50%)	256 (51%)	252 (49%)	246 (49%)	253 (49%)	254 (50%)	254 (50%)
Cough or rhinorrhea in preceding 14 days	257 (51%)	357 (70%)	301 (60%)	298 (58%)	239 (48%)	246 (48%)	187 (37%)	169 (33%)
Antibiotic use in preceding 14 days	13 (3%)	27 (5%)	39 (8%)	18 (4%)	45 (9%)	49 (10%)	48 (9%)	31 (6%)
Smoker in household	116 (23%)	136 (27%)	122 (24%)	102 (20%)	98 (20%)	117 (23%)	108 (21%)	114 (22%)
Smoker (if aged ≥15 years)	29 (11%)	37 (15%)	29 (11%)	21 (8%)	16 (7%)	21 (8%)	16 (6%)	24 (9%)
Daycare attendance (if aged <5 years)	10 (7%)	28 (18%)	18 (12%)	27 (17%)	30 (19%)	25 (17%)	44 (29%)	33 (21%)
Number of people sharing a bed*	1·8 (1-3)	1·4 (1-2)	1·4 (1-2)	1·3 (1-2)	1·4 (1-2)	1·4 (1-2)	1·5 (1-2)	1·4 (1-2)
Number of children aged <10 years in household*	1·9 (1-3)	2·6 (1-4)	2·3 (1-3)	2·2 (1-3)	2·1 (1-3)	2·5 (1-3)	2·4 (1-3)	2·4 (1-3)

Data are number (%) or *mean (IQR). Some percentages do not total 100 because of rounding.

Supplemental Table 8. Power to detect a change in non-PCV10 type invasive pneumococcal disease by age group

We performed empirical power calculation by repeatedly simulating the number of cases of NVT in each year, conditional on the midyear population, from a negative binomial distribution. For pre-vaccination years, the mean of the negative binomial distribution was the estimated incidence in the pre-vaccination period. For post-vaccination years, the mean of the negative binomial distribution was the result of increasing pre-vaccination incidence by the effect size we estimated the power for. Once the cases were simulated we refit the negative binomial regression model and compared the resulting p-value for the indicator of pre- and post-vaccination period with an alpha of 0·05. The proportion of rejected simulations (p-value<0·05) is the power. 1000 simulations were performed for each power calculation.

**Age group (years)**	**Pre-vaccination NVT IPD incidence (per 100,000)**	**Power to detect the given IRR post vaccination**					
		**Observed IRR***	**1·5**	**2·0**	**2·5**	**3·0**	**4·0**
**<5**	20·8	13·8	26·2	65·9	91·0	98·7	100·0
**5-14**	2·7	19·2	21·5	56·0	84·1	96·6	99·7
**≥15**	0·79	13·6	15·8	46·7	78·7	94·9	99·6

Abbreviations: NVT, non-PCV10 type; IPD, invasive pneumococcal disease; IRR, incidence rate ratio
*Observed IRR from data: 1·31, 1·45 and 1·47 for <5, 5-14 and ≥15 years, respectively. The power of the study was sufficient (>80%) to detect a 2·3-fold change in NVT among children <5 years.

Supplemental Figure 1. Prevalence of HIV infection among women attending ante-natal clinic at Kilifi County Hospital, 2005-2016.

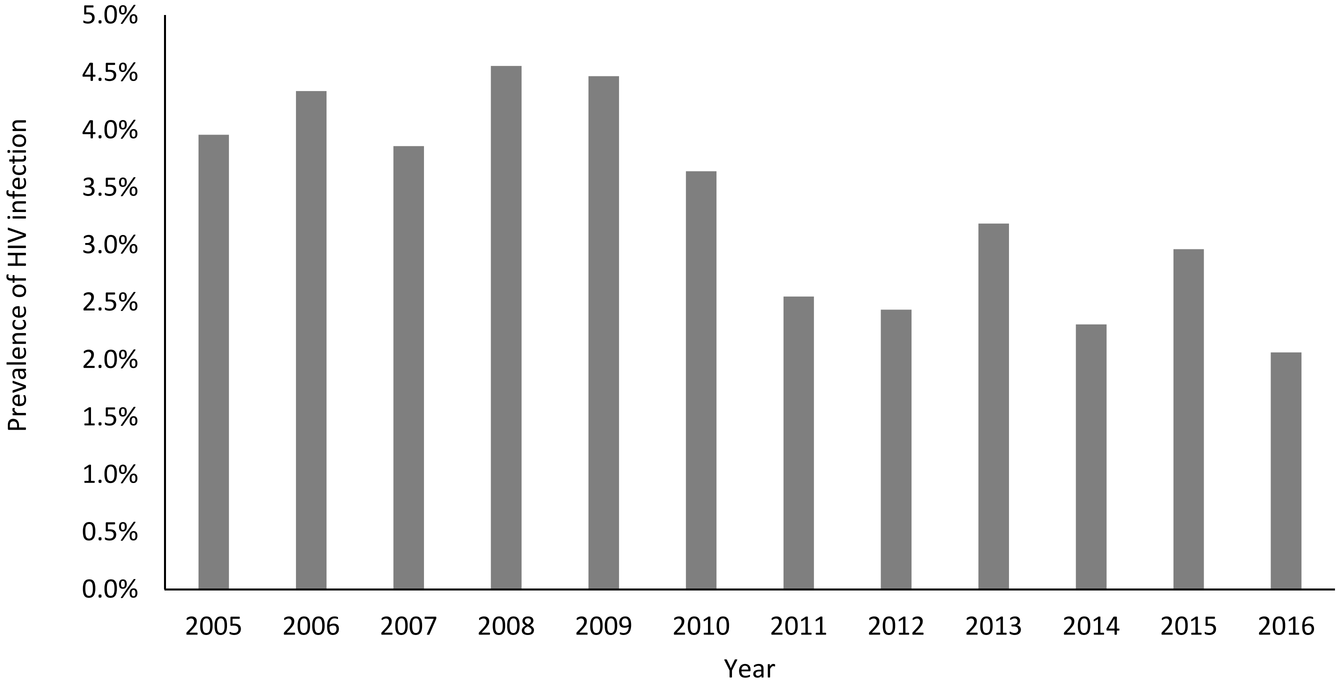

Supplemental Figure 2. Incidence of A) all admissions, B) admissions with malaria, C) admissions with moderate or severe malnutrition, and D) admissions with invasive *Staphylococcus aureus* disease, at Kilifi County Hospital among children aged <5 years in the Kilifi Health and Demographic Surveillance System, 1999-2016. Dotted vertical line indicates year of PCV10 introduction.

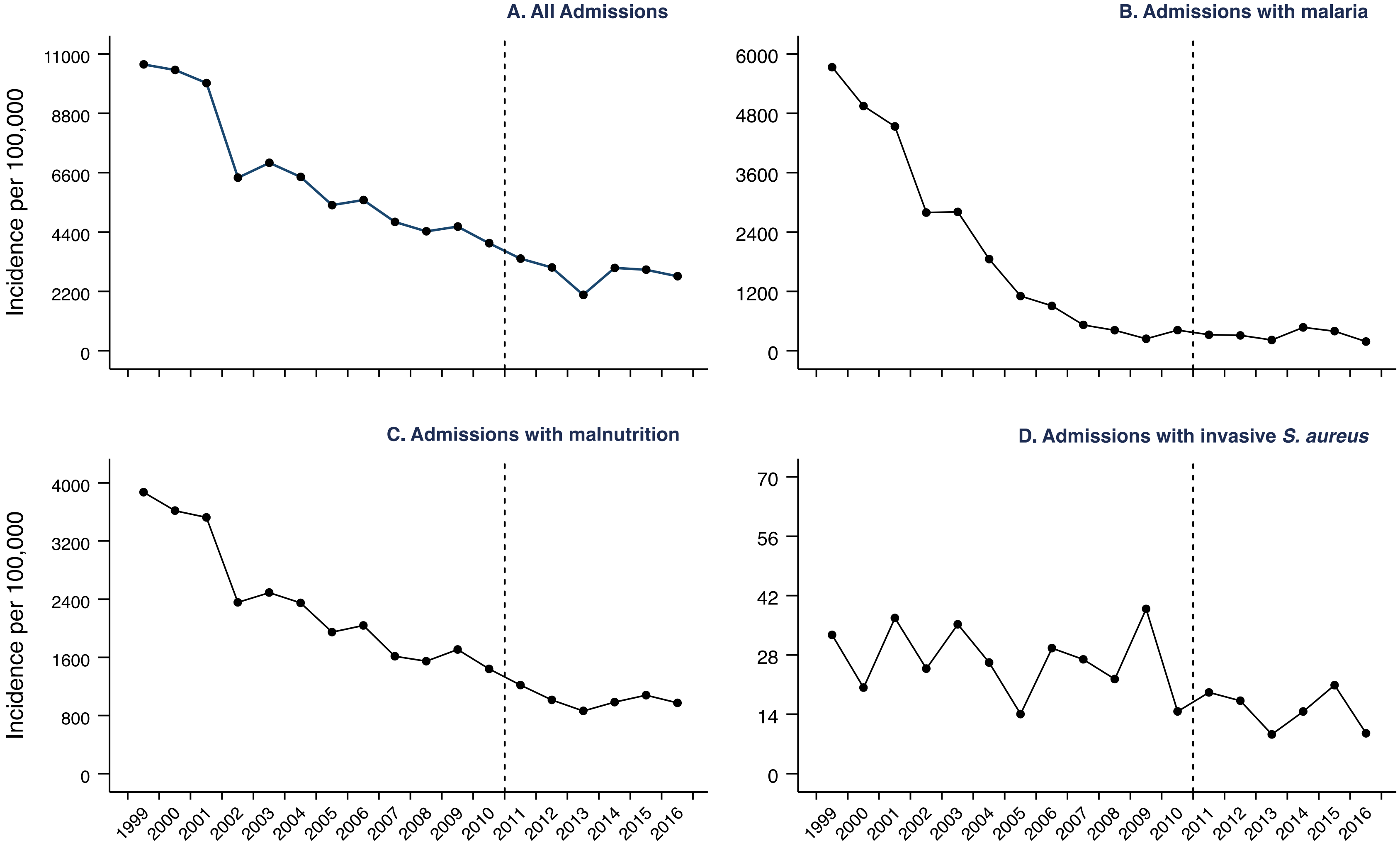

Supplemental Figure 3. Incidence of 1) all admissions, 2) admissions with malaria, 3) admissions with invasive *Staphylococcus aureus* disease at Kilifi County Hospital among individuals age 5-14 years (left) and ≥15 years (right) in the Kilifi Health and Demographic Surveillance System. Dotted vertical line indicates year of PCV10 introduction.

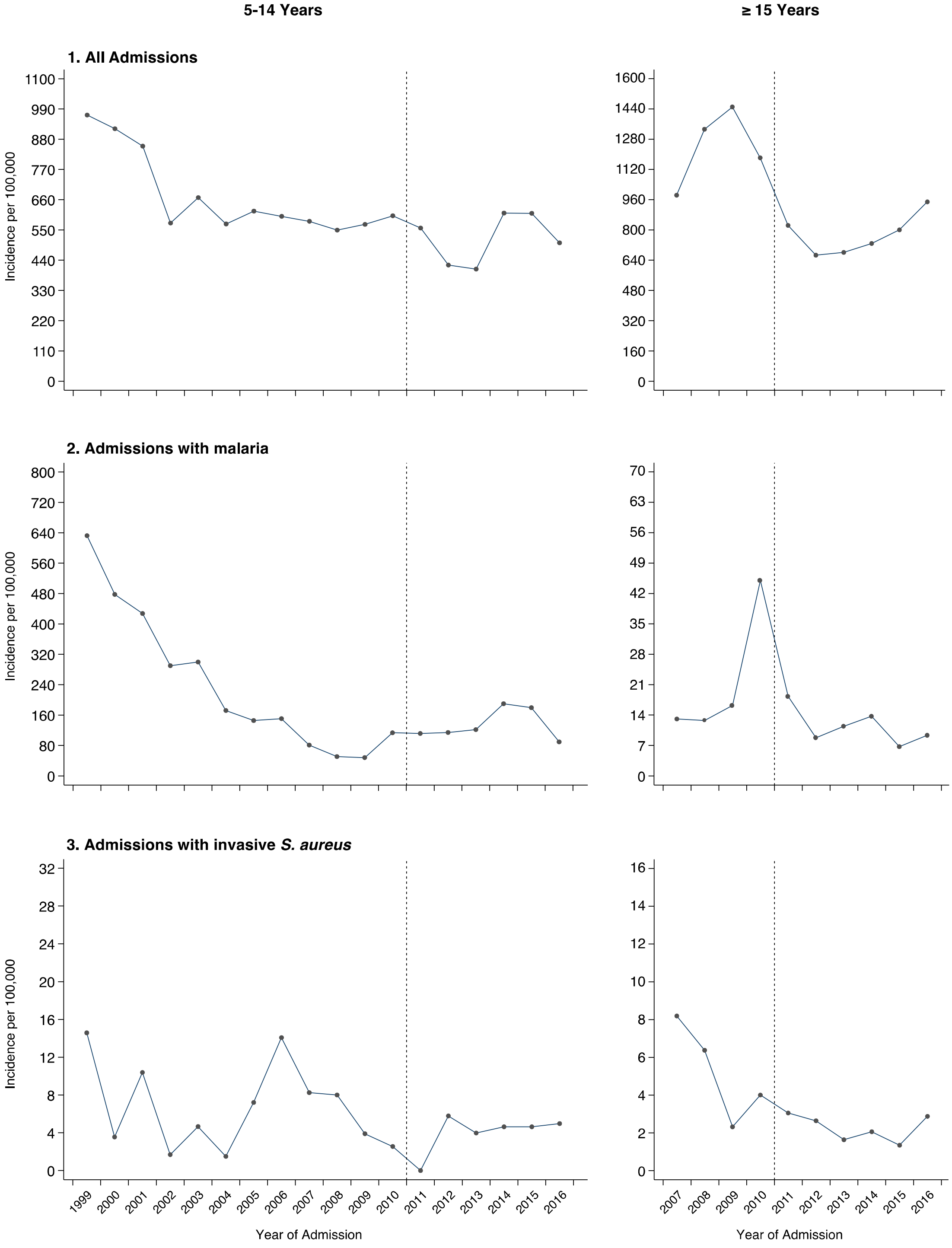

Supplemental Figure 4. Incidence of serotype 1 invasive pneumococcal disease at Kilifi County Hospital among individuals <5 years of age (black line), 5-14 years of age (red line), ≥15 years of age (blue line) in the Kilifi Health and Demographic Surveillance System. Dotted vertical line indicates year of PCV10 introduction.

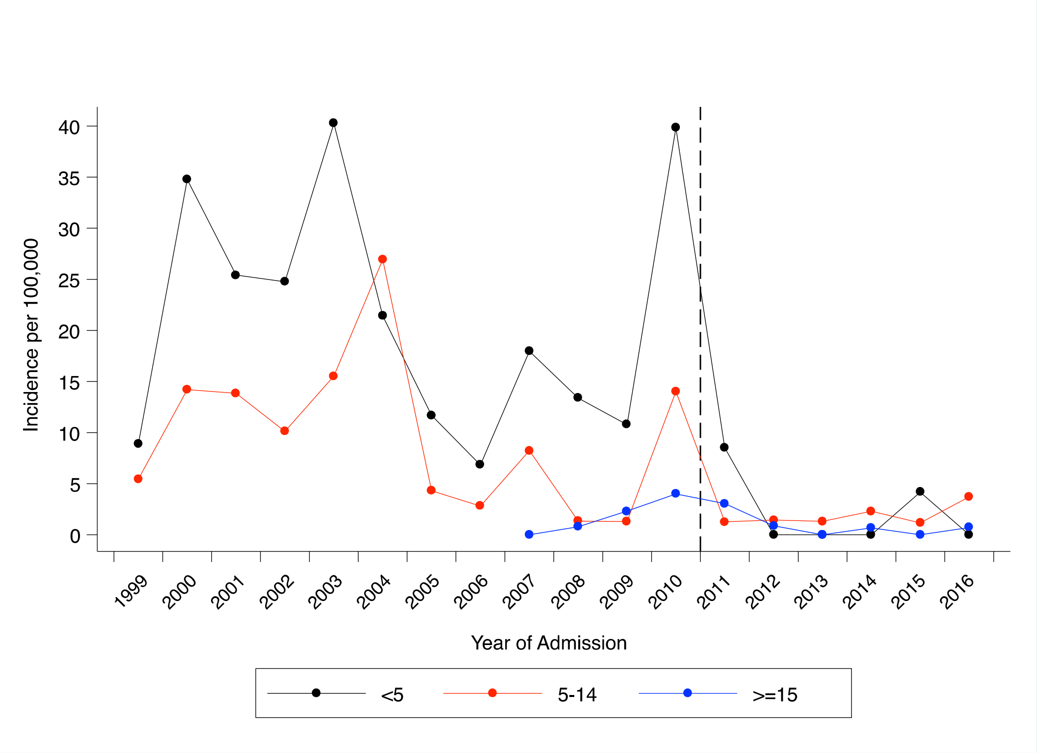

Supplemental Figure 5. Serotype-specific nasopharyngeal carriage prevalence of *Streptococcus pneumoniae* in survey participants aged <5 years, in the pre- and post-vaccine eras (restricted to serotypes carried by >1% of participants).

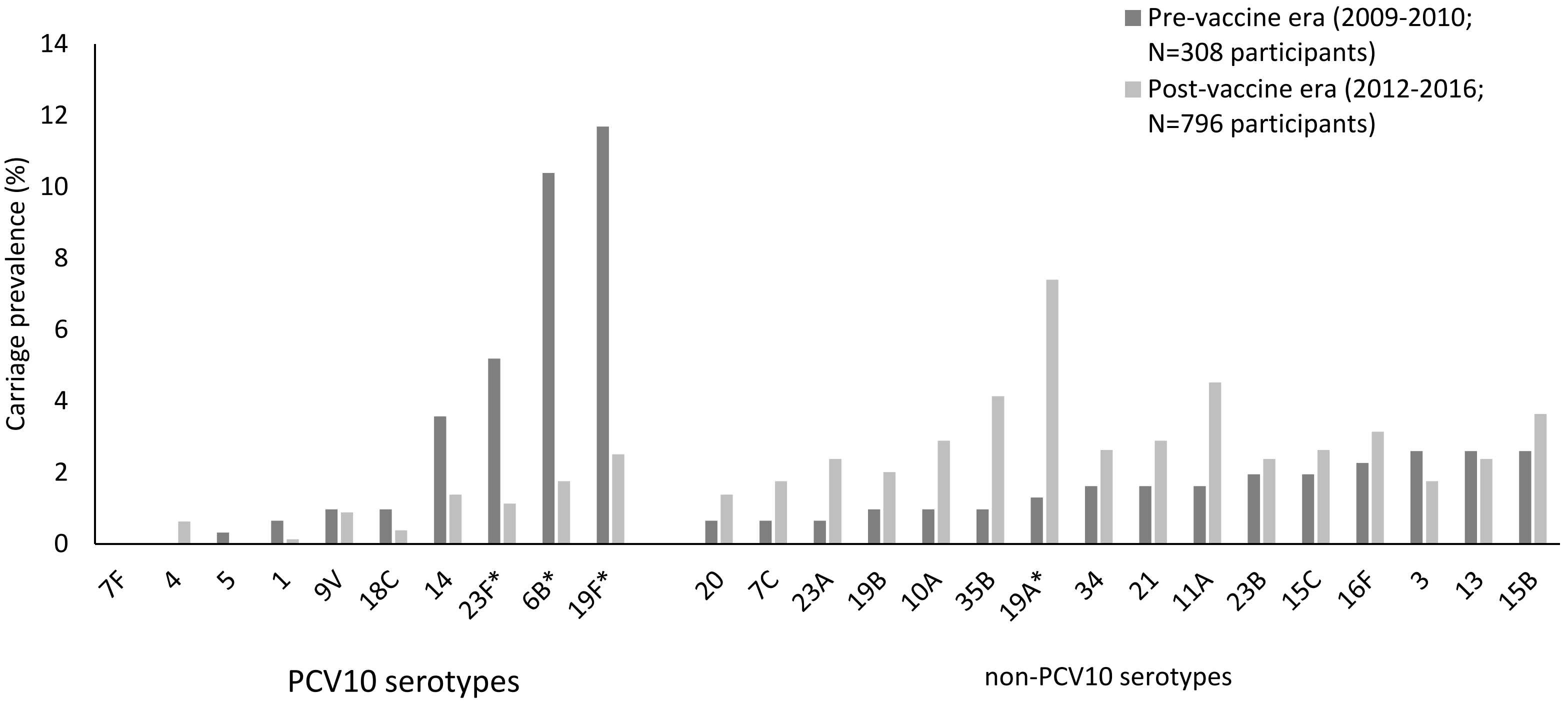

*Chi-square p-value <0·002 (corresponding to the adjustment for multiple comparisons)
In addition to the data shown, we also observed serotypes 6C (0 pre-vaccine; 1 post-vaccine), 7A (0;1), 8 (1;1), 9A (0;1), 9L (0;2), 9N (1;0), 10B (0;1), 11D (0;1), 12F (0:3), 15F (1;0), 17F (0;8), 18B (1;0), 18F (1;2), 19C (1;1), 22A (1;1), 24F (3;1), 28F (0;4), 29 (0;9), 31 (0;4), 33B (2;2), 33D (0;1), 35A (2;5), 35F (3;3), 37 (0;1), 38 (1;7),
